# Evaluation of Oxidative Stress Markers in Post-Surgical Head and Neck Cancer Patients Rehabilitated with Removable Prosthetic Restorations

**DOI:** 10.3390/antiox14111285

**Published:** 2025-10-27

**Authors:** Beata Sawczuk, Elżbieta Supruniuk, Ewa Żebrowska, Suresh Nayar, Adrian Chabowski, Teresa Sierpińska

**Affiliations:** 1 Department of Prosthetics, Medical University of Białystok, Kilińskiego 1 Street, 15-089 Bialystok, Poland; 2 Department of Physiology, Medical University of Białystok, Kilińskiego 1 Street, 15-089 Bialystok, Polandewa.zebrowska@umb.edu.pl (E.Ż.);; 3 Institute for Reconstructive Sciences in Medicine, University of Alberta, Edmonton, AB T5R 4A3, Canada

**Keywords:** oxidative stress, antioxidants, head and neck cancer

## Abstract

The effects of free radicals and chronic oxidative stress are the cause of many diseases, including those of the oral cavity, among which the most important are inflammatory processes and cancer. For this reason, an important element of the body’s defense is maintaining proper antioxidant activity. Study aim: To assess oxidative stress parameters in the saliva of patients using removable prostheses after head and neck cancer surgery. Material and methods: 44 oncological patients operated on for head and neck cancer and 20 healthy edentulous volunteers as a control group. Removable acrylic dentures were prepared for both groups. The material for oxidative stress analysis was saliva: non-stimulated saliva (NWS) and stimulated saliva (SW) after 3 months of prosthetic treatment. Results: Changes in the level of oxidative stress parameters were observed in the study group after 3 months of prosthetic treatment. Specifically, we observed a higher level of deoxyribonucleic acid (DNA)/ribonucleic acid (RNA) damage in oncology patients compared to controls. The levels of protein oxidation products—protein carbonyls, lipid peroxidation products, malondialdehyde (MDA), and nitrotyrosine—were slightly higher in the study group in all measurements. Conclusion: Based on this study, it was found that removable prostheses have a minor impact on the level of enzymatic and non-enzymatic oxidative stress parameters. This research suggests an adaptation to prosthetic restorations that results in almost restored redox balance.

## 1. Introduction

The oral cavity is an environment where numerous biochemical processes take place. This place is exposed to environmental factors, as food ingredients, strong alcohol, tobacco smoke, drugs, and microorganisms [1]. Oxygen is an essential resource for every organism. It participates in many metabolic processes and enzymatic reactions, which, among other things, convert it into reactive radicals. These are called reactive oxygen species (ROS) [2]. Free oxygen radicals are molecules containing at least one oxygen atom and at least one unpaired electron. They can function independently. They are characterized by a short lifespan but also by the remarkable ease with which they enter into chemical reactions with cellular components. From a metabolic perspective, this is not beneficial to the body due to the high degree of ROS reactivity [2]. Under physiological conditions, there is a balance between the production and accumulation of reactive oxygen species (ROS) and nitrogen species (RNS) in cells and tissues, and the capacity of the antioxidant system to eliminate them. ROS are natural by-products and, at physiological concentrations, play an important role in the proper functioning of many cellular processes. An imbalance between ROS production and antioxidant system efficiency leads to oxidative stress, which damages important cellular macromolecules, including DNA, proteins, and lipids. Oxidative stress plays a major role in the pathogenesis of salivary gland dysfunction, xerostomia, periodontitis, precancerous conditions, oral cancer, and temporomandibular joint disorders [3]. An increasing level of data indicates the involvement of ROS in the malignant transformation of cells. High ROS levels in cancer cells can lead to cellular adaptation, increased proliferation, DNA mutations, genome instability, and resistance to certain anticancer treatments, thereby facilitating cancer progression. Exposure of cells and extracellular matrix to free radicals activates multiple defense mechanisms aimed at eliminating radicals and their derivatives [4].

In recent years, there has been a growing interest in saliva-based diagnostics [5]. Measuring oxidative stress markers in saliva is currently considered an important and minimally invasive diagnostic approach, which may have diagnostic potential in the future [6]. In an attempt to describe oxidative stress, enzyme activity determination is commonly used. Superoxide dismutase (SOD) is an oxidoreductase enzyme that catalyzes the dismutation of superoxide anion radical into molecular oxygen and hydrogen peroxide. Glutathione reductase (GR) requires reduced glutathione (GSH) for its activity [4]. Catalase is another enzyme that indirectly contributes to the reduction in free radicals, but by deactivating hydrogen peroxide, it prevents its transformation into a hydroxyl radical. Uric acid (UA) exists as a major antioxidant and contributes to approximately 85% of the total antioxidant capacity of saliva [2].

In contrast to UA, glutathione (GSH) is a principal intracellular antioxidant that provides crucial cellular protection against toxic peroxides, which have a strong capacity to enhance the load of ROS. Glutathione limits this process by activating several mechanisms to work at once. These include detoxification of carcinogens, direct reduction in peroxides, and delaying the process of lipid peroxidation [5,6,7].

The continuous placement of dental prostheses potentially affects the oral microenvironment, leading to higher ROS/RNS generation to reflect local tissue stress and inflammation. Mechanical and surface properties of dentures determine their biocompatibility and chemical stability, thereby making dental appliances vulnerable to biofilm adhesion and growth [8]. Oxidative stress can also be associated with material degradation, mechanical irritation, or prosthesis-related complications such as mucosal lesions or denture stomatitis [9,10]. At present, the data to verify the above hypotheses are limited, and further studies are necessary to completely understand the interaction between prosthetic materials and salivary ROS/RNS formation. In the case of orthodontic alloys, ROS/RNS and oxidative damage were detected for stainless steel and cobalt-chromium alloys using wild-type yeast *Saccharomyces cerevisiae* model [11], while others indicate high biocompatibility of dental alloys without adverse oxidative stress [12,13]. Moreover, in studies of denture materials—pressure/heat cured acrylic resin, milled poly methyl methacrylate (PMMA), 3D printed resin, and pressure/heat cured microfilled indirect composite—no significant modifications of oxidative stress levels in saliva were observed. However, the results indicate some differences in the distribution of the values of the analyzed parameters, depending on the type of material [14]. The results of the research by Tomova et al. suggest that the presence of metal fillings significantly increases the risk of developing oxidative stress in the oral cavity [15].

To date, oxidative stress markers in saliva have not been studied in patients after surgical treatment for cancer who use prosthetic restorations. The object of the study was to evaluate the effect of the removable acrylic prostheses on the concentration of selected oxidative stress-related enzymes in both stimulated and unstimulated saliva. The measurements were performed at two time points, namely before prosthetic treatment and after three months of prosthesis use.

## 2. Materials and Methods

The study was approved by the Local Bioethics Committee of the Medical University of Bialystok (APK.002.115.2024). The study was projected, conducted, and reported according to Good Clinical Practice guidelines and the Declaration of Helsinki, with informed written consent obtained from all participants. All the recruited patients were provided with an information leaflet of the research, and informed consent was obtained by signature on a consent form.

### 2.1. Study Population

**Figure 1:**
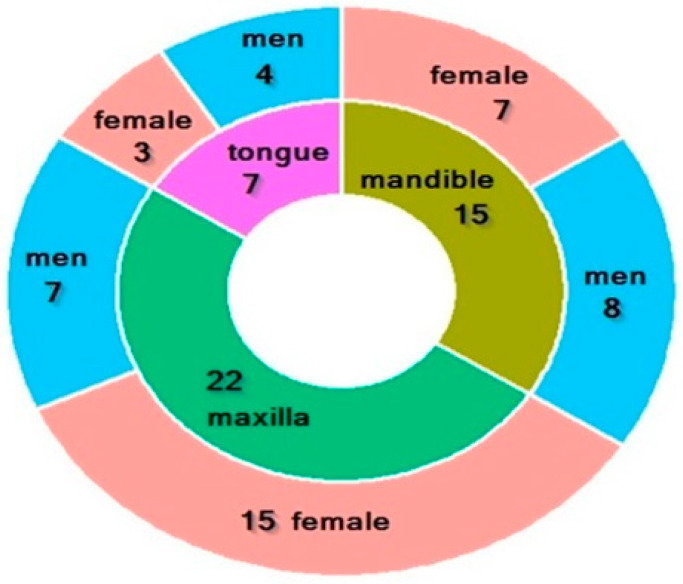
Characteristics of the study group. Characteristics of the study group. Green—22 patients with maxilla cancer (7 men, 15 women); olive—15 patients with mandible cancer (8 men, 7 women); purple—7 patients with tongue cancer (7 men, 3 women).

The study involved 64 individuals, including 39 women and 25 men. The study group consisted of 44 edentulous patients attending the Department of Prosthodontics at the Medical University of Bialystok for prosthetic rehabilitation, including 25 women and 19 men. These patients underwent surgery at the Department of Maxillofacial and Plastic Surgery of the University Clinical Hospital in Bialystok for squamous cell carcinoma of the oral cavity. Among them, 7 patients had surgery for tongue cancer, 22 for maxillary tumors, and 15 for mandibular tumors (Figure 1). The mean age of the subjects was 64.523 ± 3. Patients in the study group reported to the Prosthetics Department for prosthetic treatment at least 8 weeks after surgery.

The study included patients classified as T1 N0M0 and T2 N0M0 stages with post-operative defects classified as Type I and II according to Dreher’s classification. Dreher’s classification is terminology specific to Polish prosthodontics. It is a system for classifying a range of resected tissues that helps in selecting appropriate prosthetic restoration. It does not have an equivalent in the English language. The main cause of tooth loss in the study group was surgery and earlier tooth loss due to complications of caries and periodontal disease.

Inclusion criteria for the study group were as follows: a positive history of oral squamous cell carcinoma resection and need for prosthetic treatment, no substance addiction, and no prior use of removable prostheses.

The control group included 20 healthy, edentulous individuals who had never used removable prostheses. The mean age of the subjects was 67.4233 ± 2. Patients from the control group qualified for treatment under the National Health Funding. The main cause of tooth loss was complications of dental caries and periodontal disease. Inclusion criteria for this group were an edentulous maxilla and mandible, no previous prosthetic treatment, no substance addiction, and no systemic diseases affecting salivary composition.

Exclusion criteria for both groups included: metabolic diseases (obesity, diabetes type 1 and 2), cardiovascular diseases, systemic sclerosis, autoimmune disorders, gastrointestinal diseases, infectious diseases (HIV, HCV), chronic kidney diseases, neurological diseases, pancreatitis, immune disorders, other cancer and chronic infectious diseases, smoking, hard liquor consumption, radiotherapy, chemotherapy, and withdrawal from the study.

### 2.2. Clinical Examination

The clinical examination was conducted in two stages. The first stage included a medical interview and a dental examination. During the interview, special attention was paid to previous prosthetic treatment and the use of stimulants such as cigarettes and alcohol. Patients were also asked about oral complaints, including burning sensations, dryness, heartburn, as well as systemic diseases and medications. The oral examination was performed using dental mirror and a dental explorer under artificial lightning conditions.

The clinical examination to assess the prosthetic foundation and the need for prosthetic treatment was performed by the same physician. Oncological patients received, depending on individual indications, either upper and lower post-resection prostheses, while patients from the control group were provided with conventional complete upper and lower dentures. All clinical stages of prosthetic restoration were carried out according to generally accepted prosthodontic guidelines.

### 2.3. Saliva Collection

The studied material was NWS and SWS collected via the spitting method according to the Zalewska and Maciejczyk method [3]. All patients were off any medications for 8 h before the sampling. Saliva collection was performed in a dental office in a sitting position, with the head slightly bent downward after a 5 min adaptation period. Patient rinsed their mouth three times with distilled water. Saliva was collected into a sterile Falcon tube, which was then placed in an ice container. NWS was collected in the amount of up to 5 mL for no more than 15 min. SWS saliva was collected for 5 min. Salivary secretion was stimulated by instilling 10 μL of 2% citric acid onto the center of the tongue. Samples were measured by an automatic pipette calibrated to 0.1 mL. The saliva was immediately centrifuged (20 min, +4 °C, 5000× *g*; MPW351, MPW Med. Instruments, Warsaw, Poland) and the supernatant was frozen at −80 °C until assayed. We added an antioxidant (10 μL of 0.5 M butylated hydroxytoluene per mL of saliva supernatant) to protect the samples from oxidation. Salivary flow was calculated by dividing the saliva volume by the time required for its collection and expressed in mL/min. The study was performed before prosthetic treatment and after 3 months of prosthetic wear.

### 2.4. Redox Analysis

The analysis included the determination of enzymatic antioxidants: superoxide dismutase (SOD), catalase (CAT), glutathione reductase (GR) and non-enzymatic antioxidants: reduced glutathione (GSH), uric acid (UA), thiol. Additionally, the analysis involved the assessment of oxidative damage products, including lipid peroxidation products (MDA, HNE Adducts), protein oxidation products (proteins carbonyls), DNA/RNA oxidative damage products, and nitrosative stress biomarkers (nitrotyrosine).

Both enzymatic and non-enzymatic antioxidants, as well as oxidative damage products, were evaluated in unstimulated and stimulated saliva samples collected before prosthetic treatment and after 3 months of prosthesis use.

All spectroscopic analytical procedures followed the recommended manufacturer protocols. Total protein content in each sample was determined by the bicinchoninic acid assay method. The absorbance/fluorescence was measured using BioTek Synergy microplate reader (Winooski, VT, USA).

Superoxide Dismutase (SOD) Activity Assay Kit (CS0009; Sigma Aldrich, St. Louis, MO, USA) was based on the interaction of superoxide anions (generated by the enzyme xanthine oxidase) with the provided WST dye, yielding color at 450 nm.

Catalase Assay Kit (MAK531; Sigma Aldrich, St. Louis, MO, USA) was applied to determine catalase-mediated degradation of H_2_O_2_ using a redox dye. The change in color intensity was assessed at 570 nm.

Glutathione reductase activity was measured with the MAK535 kit (Sigma Aldrich, St. Louis, MO, USA). The method relied on the DTNB reaction with GSH generated from the reduction in GSSG by the glutathione reductase in a sample to form a yellow product (TNB2^-^). The optical density was measured using a colorimetric assay at 412 nm.

Reduced glutathione (GSH) concentration in the samples was measured with Glutathione Assay Kit (MAK517; Sigma Aldrich, St. Louis, MO, USA), and was based on the reaction with 5,5′-dithiobis (2-nitrobenzoic acid) (DTNB). The optical density was measured at 412 nm.

Fluorometric Thiol Quantitation Kit (MAK151; Sigma Aldrich, St. Louis, MO, USA) was used to evaluate the thiol content in saliva samples. The fluorescent adduct was detected with λex = 490/λem = 520 nm.

To measure uric acid concentration, QuantiChromTM Assay Kit (DIUA-250; BioAssay Systems, Hayward, CA, USA) was utilized. The method was based on the complex formation between 2,4,6-tripyridyl-s-triazine and iron in the presence of uric acid. The intensity of the color was measured at 590 nm.

Carbonyl content was determined with Protein Carbonyl Content Assay Kit (MAK094; Sigma Aldrich, St. Louis, MO, USA). The method included the derivatization of protein carbonyl groups with 2,4-dinitrophenyl-hydrazine (DNPH) and the spectrophotometric detection of stable dinitrophenyl (DNP) hydrazone adducts at 375 nm.

Lipid Peroxidation (MDA) Assay Kit (MA-MDA-2; RayBiotech, Peachtree Corners, GA) relied on the reaction of MDA with thiobarbituric acid (TBA) under high temperature (95 °C) and acidic conditions. The MDA-TBA adducts were measured at 532 nm.

HNE Adduct Competitive ELISA Kit (STA838; Cell Biolabs, San Diego, CA, USA) was used to quantify the amount of HNE protein adducts by comparison with a predetermined HNE-BSA standard curve. The absorbance was read at 532 nm.

DNA/RNA oxidative damage was determined using an ELISA kit (501130; Cayman, Ann Arbor, MI, USA). The test was based on the competition between unlabeled analyte and acetylcholinesterase (AChE) after the addition of Ellman’s reagent (with AChE substrate). The absorbance was read at 412 nm.

Nitrotyrosine content was analyzed with the K 7829 kit (Immundiagnostik, Bensheim, Germany), wherein the primary antibody–nitrated protein–peroxidase-conjugate complex was created. As a substrate, we used TMB, and absorbance was read at 450 nm.

### 2.5. Statistical Analysis of Results

The Wilcoxon non-parametric test was used for statistical analysis to compare two samples in terms of differences in their medians. A *p*-value of less than 0.05 was considered statistically significant. To compare salivary flow rates, both the Wilcoxon test and the Shapiro–Wilk test were applied. R software, version 4.5.1 (The R Foundation for Statistical Computing, Vienna, Austria, 2025) were used for all calculations. A *p*-value of less than 0.05 was considered statistically significant.

The study included all patients who met the inclusion criteria and were treated between 2019 and 2024. Due to the limited number of patients who met the criteria during this period, a total population sampling strategy was used within a single clinical center. This means that the study included the entire available population of patients eligible for the study at a given time, rather than a random sample. It should be noted that although the statistical power analysis indicates the need for a larger sample size to detect small effects. This study is observational in nature and reflects the real clinical conditions of the treatment of patients after oncological procedures at a given center.

## 3. Results

### 3.1. Unstimulated Saliva (Resting Saliva, Non-Stimulated Whole Saliva)

No statistically significant differences were observed in the amount of unstimulated saliva secreted during subsequent measurements in both the study and control groups.

**Figure 2:**
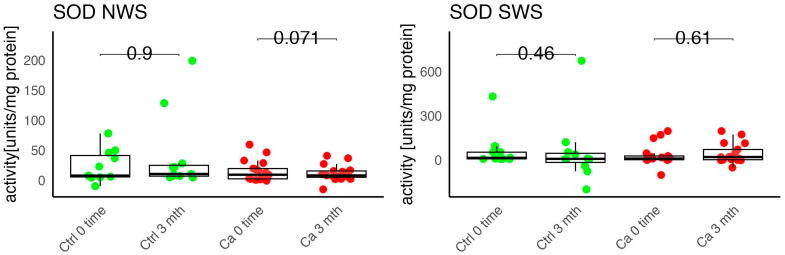
Enzymatic antioxidant activity in non-stimulated (NWS) and stimulated saliva (SWS) of cancer patients (red) and controls (green). Ca, cancer group; Ctrl, control group; SOD, superoxide dismutase.

**Figure 3:**
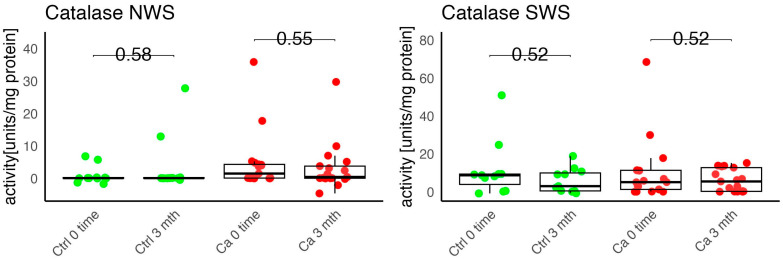
Enzymatic antioxidant activity in non-stimulated (NWS) and stimulated saliva (SWS) of cancer patients (red) and controls (green). Ca, cancer group; CAT, catalase; Ctrl, control group.

**Figure 4:**
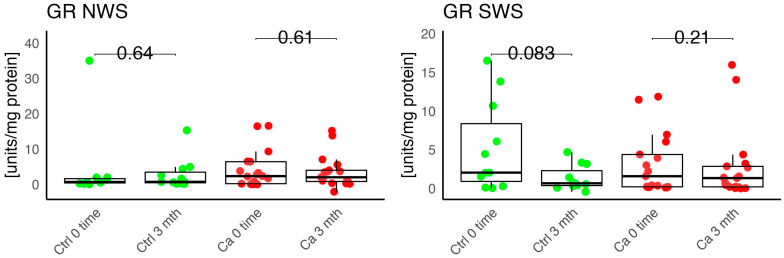
Enzymatic antioxidant activity in non-stimulated (NWS) and stimulated saliva (SWS) of cancer patients (red) and controls (green). Ca, cancer group; Ctrl, control group; GR, gluthation reductase.

**Figure 5:**
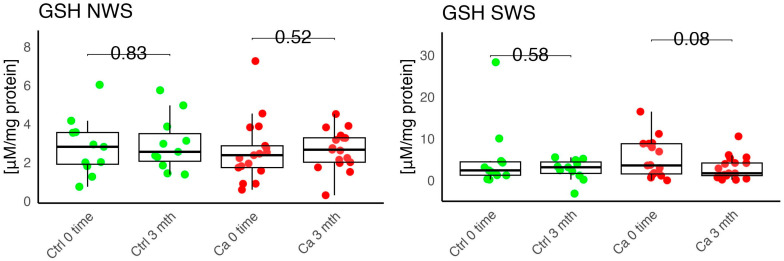
Enzymatic antioxidant in non-stimulated (NWS) and stimulated saliva (SWS) of cancer patients (red) and controls (green). Ca, cancer group; Ctrl, control group; GSH, reduced glutathione.

**Figure 6:**
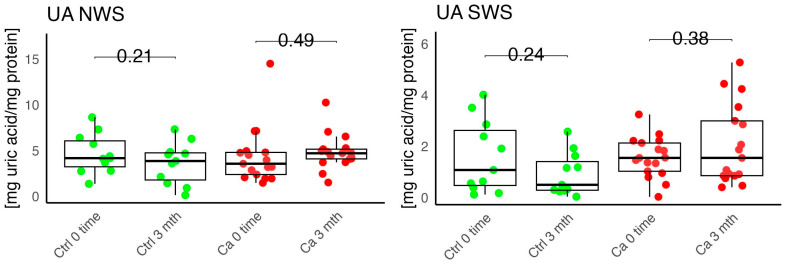
Enzymatic antioxidant concentration in non-stimulated (NWS) and stimulated saliva (SWS) of cancer patients (red) and controls (green). Ca, cancer group; Ctrl, control group; UA, uric acid.

**Figure 7:**
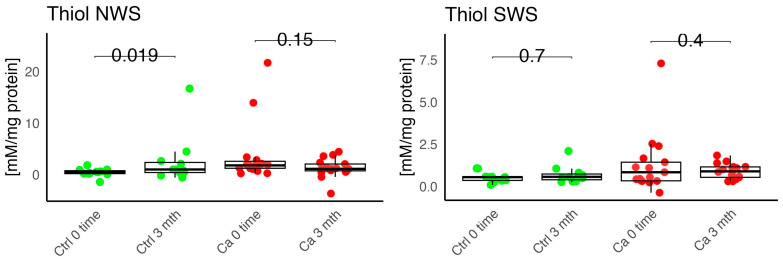
Enzymatic antioxidant, thiols, concentration in non-stimulated (NWS) and stimulated saliva (SWS) of cancer patients (red) and controls (green). Ca, cancer group; Ctrl, control group.

In the conducted study, no statistically significant differences were found in the activity of enzymatic and non-enzymatic antioxidants (SOD, CAT, GR, GSH, UA) before prosthetic treatment and after three months of prosthesis use, within both the study and control groups (Figure 2, Figure 3, Figure 4, Figure 5 and Figure 6). However, statistically significant differences were observed in thiols concentration in the control group after three months of prosthesis use (Figure 7). No statistically significant differences were found in the levels of protein oxidation products—protein carbonyls, lipid peroxidation products (MDA, HNE), DNA/RNA damage products, or nitrosative stress biomarkers (nitrotyrosine) between the groups. The activity levels of enzymatic (SOD, GR, CAT) and non-enzymatic (UA, thiols, GSH) antioxidants were higher in patients from the study group; however, these differences were not statistically significant. The levels of protein oxidation product—protein carbonyls, lipid peroxidation products—MDA and nitrotyrosine were slightly higher in the study group in all measurements.

**Figure 8:**
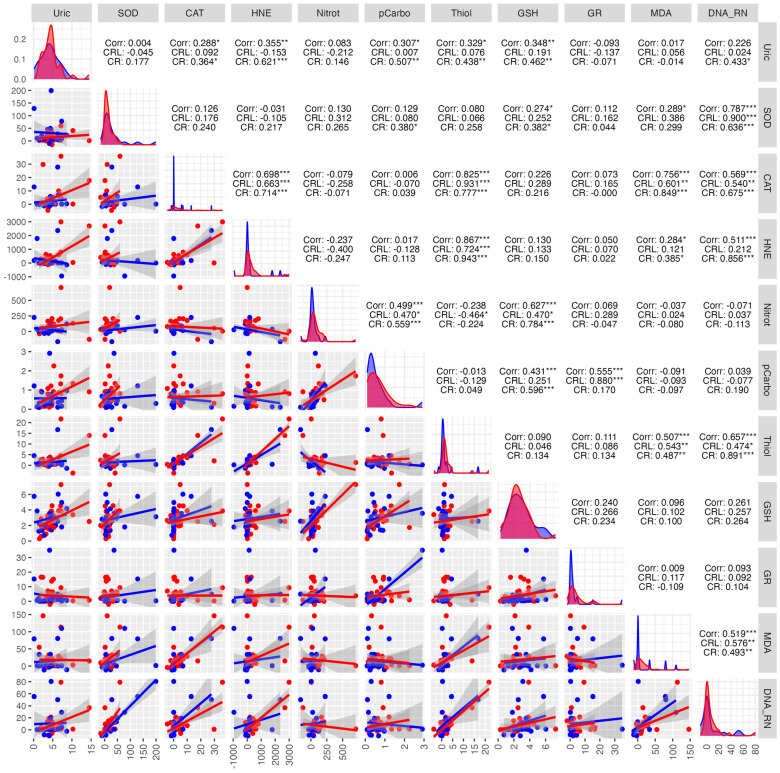
Correlations between the studied parameters in the cancer (red) and control (blue) groups in NWS. NWS—non-stimulated saliva, COR—correlation, CRL—control group, CR—cancer group, * *p* < 0.05, ** *p* < 0.01, *** *p* < 0.001, strong positive correlation, *** *p* < 0.001, strong negative correlation.

Within the study group, very strong positive correlations were observed between protein carbonyls and GSH (0.596 ***), thiol and CAT (0.777 ***), HNE and CAT (0.714 ***), HNE and MDA (0.385 *), and HNE and DNA/RNA (0.856) (Figure 8).

In the control group, very strong positive correlations were observed, indicating a high interdependence between DNA/RNA and SOD (0.900 ***), CAT and thiols (0.931 ***). The strongest negative correlations were found between nitrotyrosine and CAT (−0.258) (Figure 8).

### 3.2. Stimulated Saliva (Stimulated Whole Saliva)

No statistically significant differences were observed in the amount of stimulated saliva secretion in patients from both the study and control groups. However, a slight increase in stimulated saliva secretion was noted after three months of prosthesis use in the study group, along with a slight decrease in the control group during the same period.

The levels of enzymatic and non-enzymatic antioxidants were similar within the study and control groups before and after prosthetic treatment (Figure 2, Figure 3, Figure 4, Figure 5, Figure 6 and Figure 7). 

**Figure 9:**
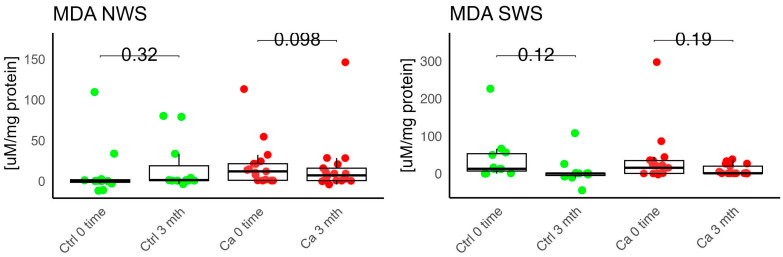
Oxidative damage product in non-stimulated (NWS) and stimulated saliva (SWS) of cancer (red) and control (green) group. Ca—cancer group, Ctrl—controls. MDA malondialdehyde.

**Figure 10:**
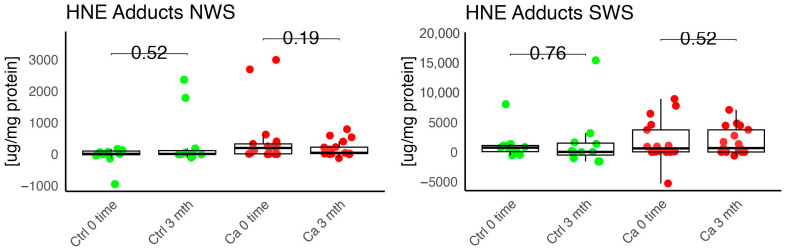
Oxidative damage product HNE Adduct in non-stimulated (NWS) and stimulated saliva (SWS) of cancer (red) and control (green) group. Ca—cancer group, Ctrl—controls.

**Figure 11:**
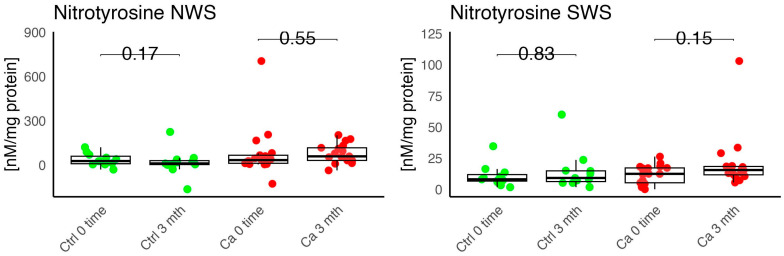
Oxidative damage product nitrotyrosine in non-stimulated (NWS) and stimulated saliva (SWS) of cancer (red) and control (green) group. Ca—cancer group, Ctrl—controls.

**Figure 12:**
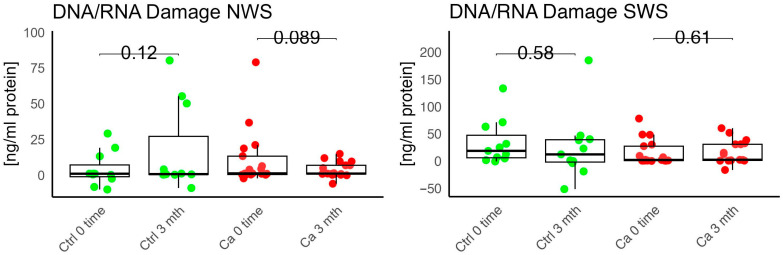
Oxidative damage product—DNA/RNA damage in non-stimulated (NWS) and stimulated saliva (SWS) saliva of cancer (red) and control (green) group. Ca—cancer group, Ctrl—controls.

**Figure 13:**
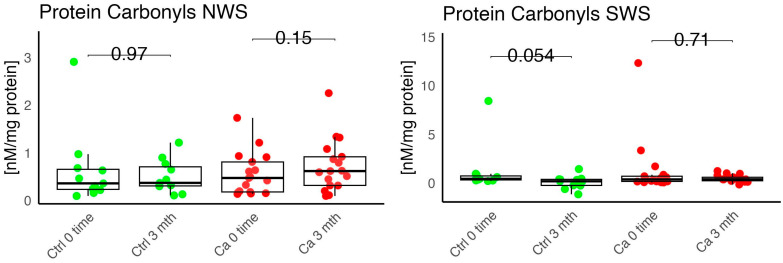
Oxidative damage product Protein carbonyls in non-stimulated (NWS) and stimulated saliva (SWS) of cancer (red) and control (green) group, Ca—cancer group, Ctrl—controls.

No statistically significant differences were observed in the levels of lipid peroxidation products (MDA, HNE), DNA/RNA damage products, or nitrotyrosine in either group (Figure 9, Figure 10, Figure 11 and Figure 12). However, the levels of protein oxidation product—protein carbonyls—were lower in the control group after three months of prosthesis use (*p* = 0.054; Figure 13).

The levels of protein oxidation product—protein carbonyls, lipid peroxidation products—MDA, nitrotyrosine, and DNA/RNA degradation products, were slightly higher in the study group in all measurements.

**Figure 14:**
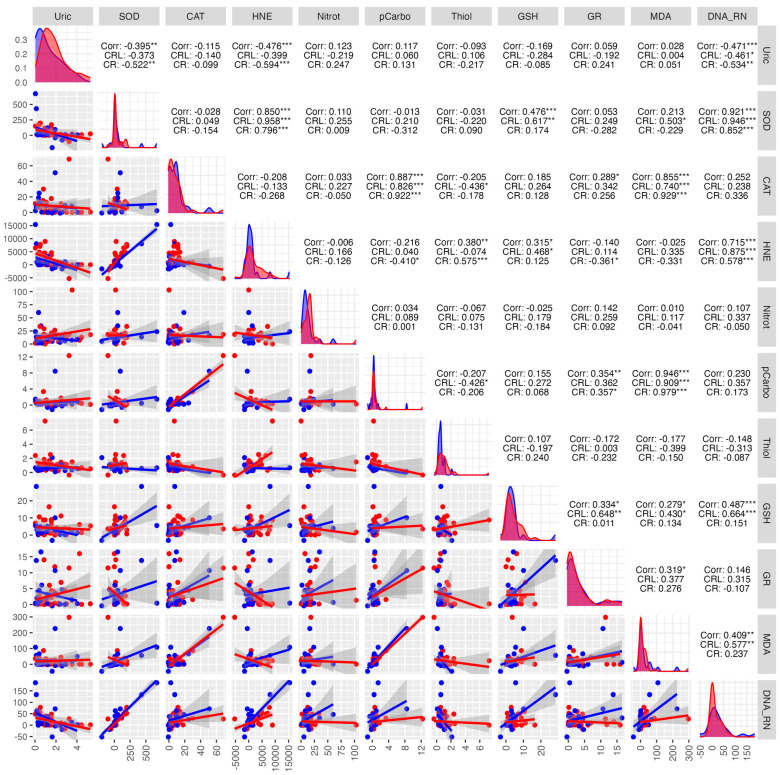
Correlations between the studied parameters in the cancer (red) and control (blue) groups in SWS. SWS—stimulated saliva, COR—correlation, CRL—control group, CR—cancer group, * *p* < 0.05, ** *p* < 0.01, *** *p* < 0.001, strong positive correlation, *** *p* < 0.001, strong negative correlation.

In the study group, a strong positive correlation was observed between protein carbonyls and CAT (0.922 ***), MDA and CAT (0.929 ***), MDA and protein carbonyls (0.979 ***), DNA/RNA damage products and SOD (0.852 ***). A strong negative correlation was found between: HNE and UA (−0.594 ***), SOD and UA (−0.522 **), DNA/RNA damage products and UA (−0.534 **) (Figure 14).

In the control group, positive correlations were observed between HNE and SOD (0.958 ***), DNA/RNA damage products and SOD (0.946 ***), MDA and protein carbonyls (0.909 ***), and DNA/RNA damage products and HNE (0.875 ***). A moderate negative correlation was found between: DNA/RNA and UA (−0.461 *), thiols and CAT (−0.436 *), thiols and protein carbonyls (−0.426 *) (Figure 14).

## 4. Discussion

Oxidative stress can be induced by various factors, including chemical compounds, radiation damage, hypoxia, ischemia, aging, and tissue damage caused by inflammatory cells. Many researchers have examined the relationship between oxidative stress parameters and periodontal disease, precancerous conditions, and oral cancers, but few studies have evaluated the impact of removable prosthesis use [7,16].

This study describes selected oxidative stress parameters in post-surgical edentulous patients using a removable prosthesis. Acrylic-based prosthetic materials are created through the polymerization of methyl methacrylate monomers, although 100% completion of polymerization is rarely achieved. The leftover of a small fraction of unreacted monomers of methyl methacrylate (MMA) is recognized as an irritant and may leach into saliva, particularly during the initial days of use [17]. The long-term effects caused by complete denture use remain unexplored. The authors hypothesized that both post-resection prosthesis and complete denture, through mechanical (pressure) and chemical (methyl methacrylate) effects, could influence oxidative stress parameters and also protein oxidation products, lipid peroxidation products, and DNA/RNA damage products.

The development of new technologies and materials science allows for the use of increasingly new materials in the manufacture of dentures. However, the impact of these materials on the oral environment is still not fully understood. The biochemical data obtained in the study by Pantea et al. indicate that incubation of saliva in the presence of the tested materials used to make temporary prostheses does not cause significant changes in the level of oxidative stress in saliva and the inflammation status of saliva, which contributes to confirming the biocompatibility of the tested temporary prosthetic materials [14]. Atay et al. investigated the effect of all-ceramic materials (such as Lava Ultimate, VITA Mark II, InCoris TZI, IPS e.max ^®^ CAD, VITA Suprinity, Cerasmart, and IPS Empress CAD) and temporary materials (such as Protemp 4, Telio CAD, CAD-Temp, Telio Lab, Temdent Classic, and Telio CS C&B) on mouse fibroblast cells. The results of this study showed that although the materials exhibited low cytotoxicity values, they could be safely used in clinical settings [18].

In the study by Birant et al., oxidative stress biomarkers decreased with dental treatment in the study group. The authors observed an increase in the level of antioxidants in saliva after prosthetic fillings and the use of protective fluoride varnish, and at the same time, a decrease in oxidative stress markers [19].

Chang et al. studied the impact of oxidative stress markers on patients’ satisfaction with removable prostheses. The authors evaluated an association between periodontal status, antioxidant levels, and prosthesis-use satisfaction. They also noticed a significant association between increased SOD levels and prosthesis-related discomfort [20]. In our study, we observed a slight increase in SOD concentration in unstimulated saliva in the study group, but the changes were not statistically significant.

In the study conducted by Boskowic et al., the highest CAT activity in patients’ saliva was observed after seven days of prosthesis use, while the greatest decrease occurred after seven days, followed by a gradual increase throughout the observation period. In our study, no statistically significant differences were found in CAT concentrations in both groups after three months of prosthesis use. Boskowic et al. also observed a peak in pro-oxidative markers, specifically MDA, after seven days of prosthesis use, which persisted for 30 days of observation [21]. This shift was related to increased levels of MDA, advanced oxidation protein products (AOPP), and nuclear factor κ-light-chain-enhancer of activated B cells (NF-κB) and decreased catalase activity [21]. The study by Reddy et al. showed that the use of a complete denture reduces the level of a psychological stress marker (SAA) and increases the level of oxidative stress indicators (TBARS, NO) [22].

Harish et al. evaluated the dependence between MDA and SOD levels and the degree of alveolar ridge resorption in edentulous patients. The authors did not observe statistically significant differences in MDA levels, whereas SOD levels showed statistical significance across all four classes. They noted an increase in MDA and SOD levels in individuals without a prosthesis, with a statistically significant difference in MDA concentration. The authors suggested that this may potentially indicate that prosthesis use results in less oxidative damage and may prevent resorption of the prosthetic foundation [23]. In our own study, we observed a slight increase in MDA concentration in the oncology group in both unstimulated and stimulated saliva compared to the control group after three months of prosthesis use. Additionally, we noted a higher GR activity in unstimulated saliva in the study group; however, this difference was not statistically significant.

The activity of oxidative stress markers was assessed not only in patients with head and neck cancer but also in those with pre-cancerous lesions. Gozanga et al. in their study on oxidative stress in patients with lichen planus found no statistically significant differences in the concentration of SOD, GSH, MDA in the saliva of people with and without lichen planus. The authors believe that this is related to the high exposure of the oral cavity environment to a number of physical, chemical, and microbiological stimuli that cause oxidative stress [24]. In a study by Singh et al., patients with lichen planus had higher MDA concentrations in the study group compared to the control group. The authors believe that the higher MDA levels in patients with oral lichen planus suggest that free radicals and the resulting oxidative damage may play a significant role in the pathogenesis of oral lichen planus lesions [25]. Vlkova et al. observed that markers of lipid peroxidation and carbonyl stress were elevated in patients with premalignant oral lesions. The reduced antioxidant status, potentially caused by decreased expression of antioxidant enzymes, may have contributed to these findings [26].

Currently, there is a lack of studies examining oxidative stress parameters in patients using removable prostheses. However, several researchers have evaluated oxidative stress markers in saliva and gingival crevicular fluid in the context of oral pathologies such as periodontitis, oral lichen planus, temporomandibular joint disorders, and smoking [27,28,29,30]. Liskmann et al. observed lower UA levels in both resting and stimulated saliva in patients with peri-implantitis compared to the control group. The authors suggest that excessive ROS production in peri-implantitis leads to an excessive oxidative stress state, which may be an important contributing factor to tissue destruction around the implant [31]. In our study, we observed higher UA levels in unstimulated saliva in the study group; however, these differences were not statistically significant. Novaković and others noted decreased SOD activity in periodontally treated patients [32].

The limitations of the study include the small number of patients, variability in the extent of surgical intervention, time elapsed since surgery, dietary differences, as well as the patients’ psycho-emotional state and their adaptation to the prostheses. Future studies should focus on more homogeneous patient groups in terms of the extent of tissue resection and consider analyzing the aforementioned parameters in patients using other types of prosthetic restorations. According to the statistical power analysis, to detect the observed difference (d = 0.17) with 80% power, 274 people would have to be recruited to each group (study group and control group). Due to the limited availability of patients who met the inclusion criteria, the group sizes were smaller, which may limit the strength of statistical inference. Nevertheless, the main strength of this research is the comprehensive assessment of multiple oxidative stress parameters that allows for any overinterpretations related to single-marker evaluation.

## 5. Conclusions

1. The study demonstrated that post-resection prostheses in cancer patients and complete dentures in healthy patients did not significantly affect the increase in selected oxidative stress parameters. The results may suggest adaptation to prostheses, reduced stress levels associated with treatment, and better nutrition.
2. The use of prostheses did not alter the amount of stimulated or unstimulated saliva in either the study or control group, which implies that wearing prostheses might not directly influence the function of salivary glands, and the dry mouth experienced by denture users could be associated with other factors such as the patient’s age, overall health conditions, or the used medications.

## Data Availability

The data presented in this study are available on request from the corresponding author.
